# Whole-Head Functional Near-Infrared Spectroscopy as an Ecological Monitoring Tool for Assessing Cortical Activity in Parkinson’s Disease Patients at Different Stages

**DOI:** 10.3390/ijms232314897

**Published:** 2022-11-28

**Authors:** Augusto Bonilauri, Francesca Sangiuliano Intra, Federica Rossetto, Francesca Borgnis, Giuseppe Baselli, Francesca Baglio

**Affiliations:** 1 Department of Electronics, Information and Bioengineering, Politecnico di Milano, 20133 Milan, Italy; 2 IRCCS Fondazione Don Carlo Gnocchi ONLUS, CADITER, 20148 Milan, Italy; 3 Faculty of Education, Free University of Bolzano-Bozen, 39042 Brixen, Italy

**Keywords:** continuous wave functional near-infrared spectroscopy, rehabilitation monitoring, brain activation mapping, motor tasks, functional near-infrared signal processing, Parkinson Disease, hemispheric hemodynamic response, clinical fNIRS translation

## Abstract

Functional near-infrared spectroscopy (fNIRS) is increasingly employed as an ecological neuroimaging technique in assessing age-related chronic neurological disorders, such as Parkinson’s disease (PD), mainly providing a cross-sectional characterization of clinical phenotypes in ecological settings. Current fNIRS studies in PD have investigated the effects of motor and non-motor impairment on cortical activity during gait and postural stability tasks, but no study has employed fNIRS as an ecological neuroimaging tool to assess PD at different stages. Therefore, in this work, we sought to investigate the cortical activity of PD patients during a motor grasping task and its relationship with both the staging of the pathology and its clinical variables. This study considered 39 PD patients (age 69.0 ± 7.64, 38 right-handed), subdivided into two groups at different stages by the Hoehn and Yahr (HY) scale: early PD (ePD; N = 13, HY = [1; 1.5]) and moderate PD (mPD; N = 26, HY = [2; 2.5; 3]). We employed a whole-head fNIRS system with 102 measurement channels to monitor brain activity. Group-level activation maps and region of interest (ROI) analysis were computed for ePD, mPD, and ePD vs. mPD contrasts. A ROI-based correlation analysis was also performed with respect to contrasted subject-level fNIRS data, focusing on age, a Cognitive Reserve Index questionnaire (CRIQ), disease duration, the Unified Parkinson’s Disease Rating Scale (UPDRS), and performances in the Stroop Color and Word (SCW) test. We observed group differences in age, disease duration, and the UPDRS, while no significant differences were found for CRIQ or SCW scores. Group-level activation maps revealed that the ePD group presented higher activation in motor and occipital areas than the mPD group, while the inverse trend was found in frontal areas. Significant correlations with CRIQ, disease duration, the UPDRS, and the SCW were mostly found in non-motor areas. The results are in line with current fNIRS and functional and anatomical MRI scientific literature suggesting that non-motor areas—primarily the prefrontal cortex area—provide a compensation mechanism for PD motor impairment. fNIRS may serve as a viable support for the longitudinal assessment of therapeutic and rehabilitation procedures, and define new prodromal, low-cost, and ecological biomarkers of disease progression.

## 1. Introduction

In recent decades, functional near-infrared spectroscopy (fNIRS) has received increasing interest for non-invasively assessing cortical hemodynamic activity [1]. This optical imaging technique allows the direct measurement of either variations in (i.e., $\Delta \left[{\mathrm{HbO}}_{2}\right]$ and Δ[HbR]) or absolute concentrations of oxygenated ${\mathrm{HbO}}_{2}$ and deoxygenated (i.e., reduced) hemoglobin HbR in the brain [2], thereby granting useful insights regarding neurovascular coupling (i.e., interaction mechanisms between neuronal activity and cortical perfusion) and localized hemodynamic responses [3]. Specifically, this technique employs pairs of sources and detectors of NIR-light placed onto the subject’s scalp through a traditional electroencephalography (EEG) cap to measure the absorbances of at least two NIR-wavelengths around the isosbestic point of 805 nm. The absorbances are converted into ${\mathrm{HbO}}_{2}$ and HbR concentrations, according to modified Beer–Lambert law [4], due to other major components of biological tissues being almost negligible within the NIR spectral region.

Compared to functional Magnetic Resonance Imaging (fMRI)—which is regarded as the gold standard for functional neuroimaging—the fNIRS presents several practical advantages. In particular, this technique facilitates the performance of low-cost functional measurements of cortical activity, with a higher temporal resolution in an open environment, without interfering with diagnostic/therapeutic procedures or causing excessive discomfort to the subject [5]. In addition, the ecological environment in which the functional measurements are performed allows the implementation of different typologies of functional tasks, such as motor, walking, somatosensory, and cognitive tasks [6,7]. Finally, fNIRS measurements are less sensitive to motion artifacts, as they are bounded only to the coupling between a subject’s scalp and the fNIRS cap. As a result, the measurements can be performed with poorly cooperating subjects, people suffering from claustrophobia, or people with metal implants. Nevertheless, the practical benefits of fNIRS promote its multimodal integration with other neuroimaging modalities, such as EEG [8] or even post-fusion imaging with anatomical MRI, to enhance the localization of functional activation [9,10]. Indeed, fNIRS presents a limited spatial resolution for source-detector distances, thereby reducing its imaging capabilities to cortical surface activity only [11] and confining this technique primarily to research applications. However, the practical advantages of fNIRS, together with its ability to perform multiple longitudinal acquisitions for monitoring brain activity and neuroplasticity, have increased its relevance in clinical and therapeutic applications, such as stroke recovery [12], brain-computer interfaces, and neurofeedback [5,13].

The fNIRS technique is increasingly employed in age-related chronic neurological disorders to provide a cross-sectional characterization of such pathologies in ecological settings [14]. In particular, fNIRS has attracted increasing interest in assessing Parkinson’s disease, due its practical advantages in investigating prefrontal cortex (PFC) activity during gait, postural stability, and dual-task walking conditions, thereby determining motor vs. non-motor effects and executive dysfunction due to the pathology.

More specifically, actual applications investigated the effects of freezing of gait (FoG) and/or its interaction with anti-Parkinsonian medications [15,16,17,18,19], as well as the variations in PFC activation levels due to aforementioned tasks [20,21,22,23,24,25,26,27,28,29,30,31]. Other studies have also investigated the effects of deep brain stimulation on PFC oxygenation and activation [32,33], premotor cortex (PMC) activation [34], map PFC [35] and parietal-occipital functional connectivity [36], map resting-state networks, and responses in the temporal-occipital cortex to auditory tasks [37].

Moreover, the practical advantages of fNIRS make this technique suitable for characterization over time on how hemodynamic activity changes as the PD stages progress. Indeed, in standard clinical practices, a neuroimaging assessment with MRI and/or fMRI is performed only at the time of diagnosis, to verify the absence of additional comorbidities [38]. Then, despite the high relevance of a longitudinal assessment, such an assessment is often overlooked due to the complexity of the prognosis associated with the pathology [39].

However, there is a lack of studies assessing cortical activation in relation to the stages of PD. Therefore, in this work, we sought to investigate cortical activity of PD patients during motor-grasping tasks and the relationship of such activity with both the stage of the pathology and clinical variables. This approach first required us to establish a clear separation of two subgroups of PD participants based on the stage of the pathology: early PD (ePD) patients and moderate PD (mPD) patients, based on the Hoehn and Yahr scale (HY). Then, we computed group-level fNIRS statistical activation maps to compare the groups individually and simultaneously (i.e., interaction between the groups and the task conditions). Region of Interest (ROI) analysis was also employed to support the results obtained from group-level maps and to assess the Spearman’s correlation of fNIRS data with clinical variables.

## 2. Results

### 2.1. Patient Data Characterization

Significant differences in the characteristics of the ePD group and the mPD group and in the clinical variables are presented in Table 1. The data show group differences in age, disease duration, and the Unified Parkinson’s Disease Rating Scale (UPDRS), while no significant differences were found for the Cognitive Reserve Index questionnaire (CRIQ), the Stroop Color and Word error score (SCWE), or the Stroop Color and Word time score (SCWT). The first result confirms that a higher stage of PD is associated with higher motor impairment and progression of the pathology through time, while the subsequent results indicate that the subgroups did not differ in terms of cognitive reserve and performance in a well-established cognitive test. Significant differences in the 5-level EQ5D scale (EQ5D5L) also indicated that higher levels of PD severity are associated with a lower level of perceived health status.

### 2.2. Group-Level Activation Maps

**Figure 1:**
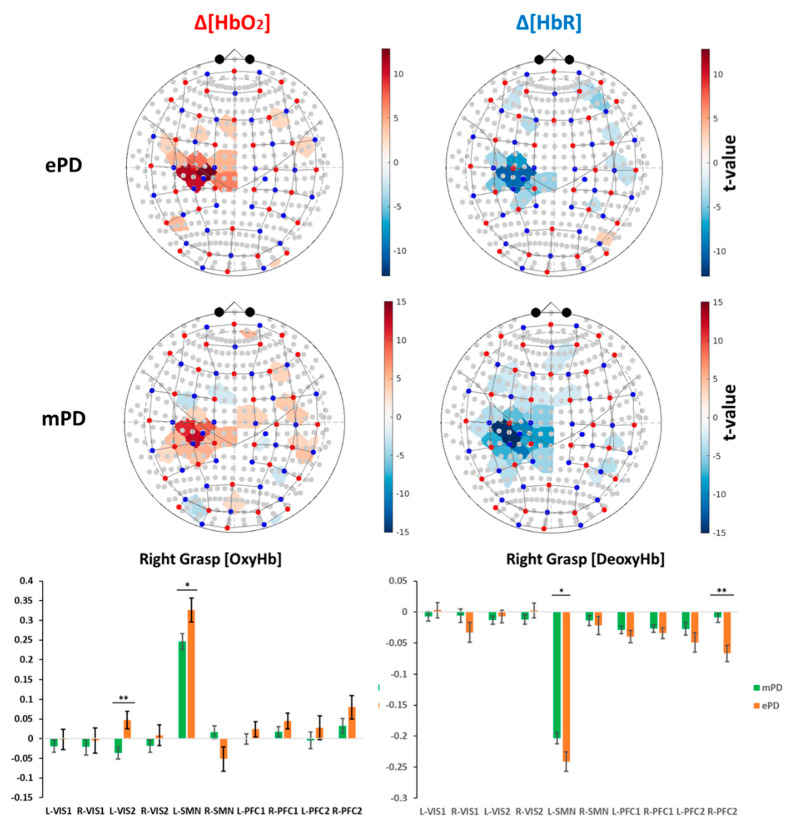
(**Top**) Significant group-level activation maps referring to right-grasp task. Results were corrected for multiple comparisons (*p_FDR_* < 0.05). (**Bottom**) ROI analysis of group-level right-grasp task for the ePD and mPD groups: (*) significant contrasts not corrected for multiple comparisons (*p* < 0.05); (**) significant contrasts corrected for multiple comparisons (*p_FDR_* < 0.05). The correspondence between ROIs (i.e., horizontal axis) with functional and anatomical cortical areas is reported in Section 4.6.

**Figure 2:**
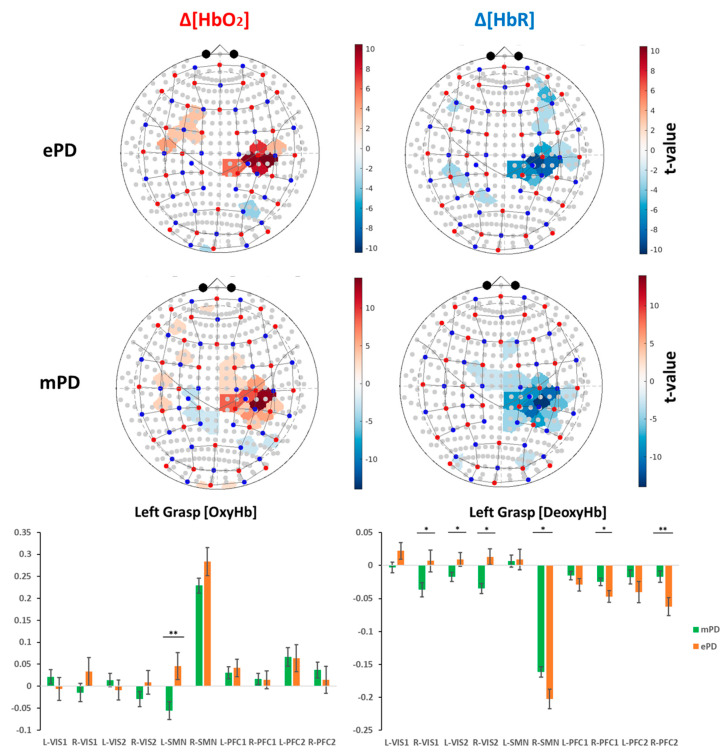
(**Top**) Significant group-level activation maps referring to left-grasp task. Results were corrected for multiple comparisons ($p_{FDR}<0.05$). (**Bottom**) ROI analysis of group-level left-grasp task for the ePD and mPD groups: (*) significant contrasts not corrected for multiple comparisons (p<0.05); (**) significant contrasts corrected for multiple comparisons ($p_{FDR}<0.05$). The correspondence between ROIs (i.e., horizontal axis) with functional and anatomical cortical areas is reported in Section 4.6.

Group-level activation maps across chromophores and task conditions for the ePD and mPD groups are separately represented in Figure 1 and Figure 2, while ROI-based significant activations are reported in Table 2. In Section 4.6, we report the list of selected ROIs and their correspondence with functional and anatomical cortical areas.

From these results, we concluded, as expected for both $\Delta \left[{\mathrm{HbO}}_{2}\right]$ and Δ[HbR], that contralateral activations were the most significant ones; i.e., the left (L-SMN) and right (R-SMN) motor areas due to right-grasp (RG) and left-grasp (LG) tasks, respectively. A significant ipsilateral deactivation of the L-SMN due to LG was also found for the mPD group (t = −2.784, *p* = 0.007). In addition, we found that L-VIS2 for $\Delta \left[{\mathrm{HbO}}_{2}\right]$-RG, L-PFC2 for $\Delta \left[{\mathrm{HbO}}_{2}\right]$-LG and Δ[HbR]-RG, L-PFC1 for $\Delta \left[{\mathrm{HbO}}_{2}\right]$-LG, Δ[HbR]-LG, and Δ[HbR]-RG were major non-motor regions that presented significant ancillary activation. Isolated significant activations were found in the ePD group in R-VIS1 for Δ[HbR]-RG (t = −2.046, *p* = 0.043), L-PFC2 (t = −2.510, *p* = 0.013) and R-PFC2 (t = −4.678, *p* < 0.001) for Δ[HbR]-LG, R-PFC2 for Δ[HbR]-RG (t = −5.069, *p* < 0.001), R-PFC2 (t = 2.653, *p* = 0.009), and R-PFC1 (t = 2.253, *p* = 0.026) for $\Delta \left[{\mathrm{HbO}}_{2}\right]$-RG. In contrast, significant isolated activations were found in the mPD group in R-PFC2 for $\Delta \left[{\mathrm{HbO}}_{2}\right]$-LG (t = 2.024, *p* = 0.045), L-VIS1 (t = −3.348, *p* = 0.001), and L-VIS2 (t = −2.518, *p* = 0.013) for Δ[HbR]-LG.

**Figure 3:**
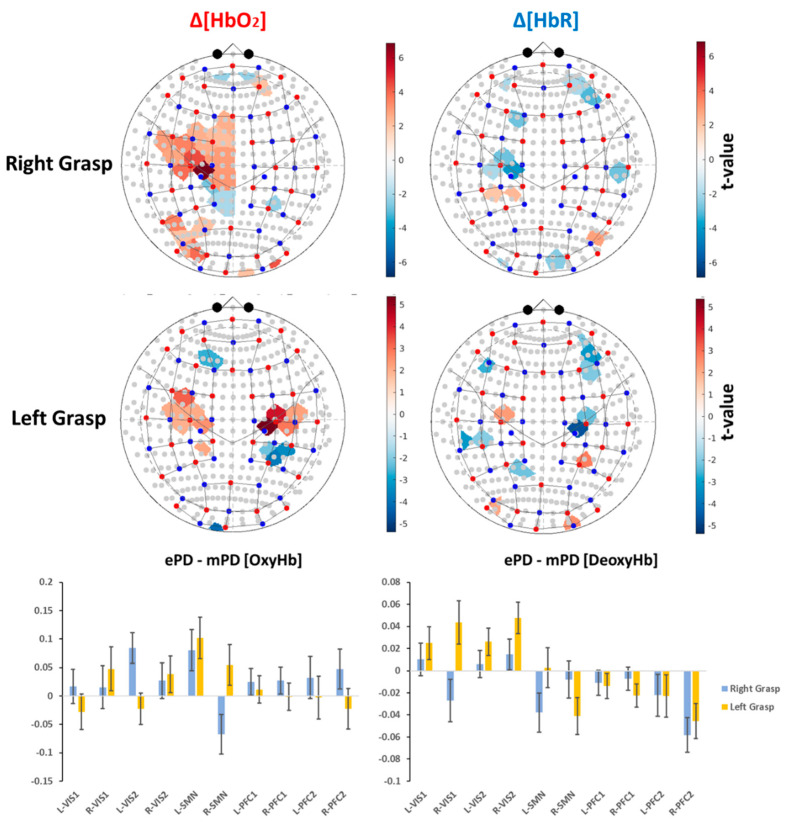
(**Top**) Significant group-level contrast activation maps for the ePD and mPD groups. Results were not corrected for multiple comparisons (p<0.05). Positive $t_{value}$ indicates a greater activation in the ePD group than in the mPD group.

The ePD and mPD activation maps have been separately represented in Figure 1 and Figure 2, and significant differences were analyzed, as shown in Table 1. Next, the mapping of ePD vs. mPD contrasts were analyzed. Obviously, the overall mapping similarities and the emphasis of motor areas were canceled, as only the contrasts were represented. Nevertheless, the patterns of significant differences were outlined and discussed. The contrast maps were extracted by a two-tailed *t*-test contrast, regarding task condition vs. group interactions (Figure 3 and Table 3).

We found a unilateral hemispheric difference in both $\Delta \left[{\mathrm{HbO}}_{2}\right]$ (L-SMN: t = 2.228, *p* = 0.027) and Δ[HbR] activity (L-SMN: t = −2.120, *p* = 0.036) due to RG, meaning that the ePD group presented a greater contralateral activation in RG compared to the mPD group. Conversely, LG caused a significant $\Delta \left[{\mathrm{HbO}}_{2}\right]$ difference in the ipsilateral left motor areas (L-SMN: t = 2.768, *p* = 0.006), which was counter-intuitive, as we expected to find this difference in the contralateral hemisphere was due to the hand performing the task. However, Figure 3 suggests a difference in the contralateral hemisphere due to LG, and ROI analysis revealed that there was only a slight tendency toward significance (R-SMN: t = 1.516, *p* = 0.132), as a comparable number of positive vs. negative t-value channels were averaged simultaneously.

Concerning the contrast between the ePD group and the mPD group in non-motor areas, most of differences were found for the LG condition and the Δ[HbR] chromophore instead of the RG condition and $\Delta \left[{\mathrm{HbO}}_{2}\right]$. In summary, the ePD group presented a greater activation in both R-VIS1 (t = 2.223, *p* = 0.028 for Δ[HbR]-LG) and bilateral VIS2 (L-VIS2: t = 2.112, *p* = 0.036 for Δ[HbR]-LG, t = 3.129, *p* = 0.002 for $\Delta \left[{\mathrm{HbO}}_{2}\right]$-RG; R-VIS2: t = 3.377, *p* = 0.001 for Δ[HbR]-LG). In contrast, the mPD group presented a greater activation in R-PFC1 (t = −2.119, *p* = 0.036 for Δ[HbR]-LG) and R-PFC2 (t = −2.880, *p* = 0.005 for Δ[HbR]-LG; t = −3.706, *p* < 0.001 for Δ[HbR]-RG). Further details regarding significant group-level contrasts among the ePD, mPD, and ePD vs. mPD subdividisons c across ROIs, task conditions, and chromophores are provided in supplementary data (sheet *groupAnalysisEPD*, *groupAnalysisMPD*, and *groupAnalysisEPDvsMPD*, respectively).

### 2.3. ROI-Based Correlation Analysis

The last analysis involved the ROI-based correlation analysis (ROI-CA) between subject-level fNIRS data (i.e., beta-GLM coefficients) and clinical variables. Table 4 provides a summary of all combinations of ROI, grasping tasks (LG or RG), and chromophores (Δ[HbR] or $\Delta \left[{\mathrm{HbO}}_{2}\right]$) that presented at least one significant correlation with any clinical variable. No significant difference was found for LG and $\Delta \left[{\mathrm{HbO}}_{2}\right]$ combinations and, accordingly, no line is reported in Table 4. Further details regarding the full tables of correlations and partial correlations are provided in supplementary data (sheet *correlationAnalysis* and *partialCorrelationAnalysis*).

It is worth remarking that the opposite sign of $\Delta \left[{\mathrm{HbO}}_{2}\right]$ and Δ[HbR] is also replicated in the opposite signs of the correlation coefficients. Therefore, activation increments with the regressed index are indicated by a positive correlation in $\Delta \left[{\mathrm{HbO}}_{2}\right]$ or by a negative correlation of Δ[HbR] (from negative values to less negative values). In the following discussion, significant correlations of either $\Delta \left[{\mathrm{HbO}}_{2}\right]$ or (more frequently) Δ[HbR] are discussed.

Age provided the highest number of significant correlations, even if all referred to Δ[HbR] cases. In particular, age was significantly correlated with cortical activity, due to Δ[HbR]-RG over L-SMN (Spearman’s correlation coefficient $\rho_{S}$ = 0.441, *p* = 0.005), R-PFC2 ($\rho_{S}$ = 0.394, *p* = 0.013), and R-PFC1 ($\rho_{S}$ = 0.329, *p* = 0.41). In addition, age was significantly correlated with Δ[HbR]-LG over L-VIS2 ($\rho_{S}$ = −0.366, *p* = 0.022), R-SMN ($\rho_{S}$ = 0.345, *p* = 0.031), and L-PFC2 ($\rho_{S}$ = 0.456, *p* = 0.004).

It is worth noting that age across ROIs and tasks was always positively correlated with Δ[HbR] in motor areas that were contralateral to the hand performing the task, positively correlated with the frontal cortex, and negatively correlated with the visual cortex. As noted above, a positive $\rho_{S}$-coefficient for Δ[HbR] provides evidence of lower activation with age. Congruently, a negative $\rho_{S}$ for $\Delta \left[{\mathrm{HbO}}_{2}\right]$ was found, although it was not statistically significant ($\rho_{S}$ = −0.185 *p* = 0.259 for $\Delta \left[{\mathrm{HbO}}_{2}\right]$-RG over L-SMN; $\rho_{S}$ = −0.3, *p* = 0.063 for $\Delta \left[{\mathrm{HbO}}_{2}\right]$-LG over R-SMN).

Given the age dependency and the unbalanced ages in the ePD and mPD groups, we decided to investigate other variables according to partial correlation, using age as the covariate and noting which variables remained statistically significant (Table 5) after the separation of aging effects. Interestingly, the only clinical variable that was significantly correlated with motor areas was the CRIQ ($\rho_{S}$ = −0.531, *p* < 0.001 for Δ[HbR]-LG; partial correlation $\rho_{\mathrm{Spart}}$ = −0.515, *p <* 0.001). Conversely, other clinical variables were significantly correlated with both frontal and occipital areas. Specifically, disease duration was negatively correlated with $\Delta \left[{\mathrm{HbO}}_{2}\right]$-RG over R-VIS1 ($\rho_{S}$ = 0–0.397, *p* = 0.012; partial correlation coefficient $\rho_{\mathrm{Spart}}$ = −0.41, *p* = 0.011) and R-VIS2 ($\rho_{S}$ = −0.366, *p* = 0.022; $\rho_{\mathrm{Spart}}$ = −0.355, *p* = 0.029). In addition, the UPDRS was negatively correlated to $\Delta \left[{\mathrm{HbO}}_{2}\right]$-RG over R-VIS2 ($\rho_{S}$ = −0.388, *p* = 0.015; $\rho_{\mathrm{Spart}}$ = −0.363, *p* = 0.025) together with Δ[HbR]-LG over L-PFC2 ($\rho_{S}$ = 0.355, *p* = 0.037). Partial correlation of the UPDRS with Δ[HbR]-LG was not maintained ($\rho_{\mathrm{Spart}}$ = 0.131, *p* = 0.433).

Finally, Stroop test scores were correlated with fNIRS data almost exclusively over bilateral PFC1 and PFC2, as expected. No other significant correlation was found, except for Δ[HbR]-RG over R-VIS1 ($\rho_{S}$ = 0.371, *p* = 0.024; $\rho_{\mathrm{Spart}}$ = 0.347, *p* = 0.038). SCWE score was correlated only with Δ[HbR]-RG over R-PFC2 ($\rho_{S}$ = 0.539, *p <* 0.001; $\rho_{\mathrm{Spart}}$ = 0.457, *p* = 0.005) and R-PFC1 ($\rho_{S}$ = 0.464, *p* = 0.004; $\rho_{\mathrm{Spart}}$ = 0.382, *p* = 0.022). Conversely, SCWT was positively correlated with L-PFC2 ($\rho_{S}$ = 0.476, *p* = 0.003; $\rho_{\mathrm{Spart}}$ = 0.448, *p* = 0.007) and L-PFC1 ($\rho_{S}$ = 0.354, *p* = 0.034; no significant partial correlation) for Δ[HbR]-RG, while with R-PFC2 ($\rho_{S}$ = 0.394, *p* = 0.017; $\rho_{\mathrm{Spart}}$ = 0.381, *p* = 0.024) and R-PFC1 ($\rho_{S}$ = 0.398, *p* = 0.016; $\rho_{\mathrm{Spart}}$ = 0.399, *p* = 0.018) for Δ[HbR]-LG. Again, it is worth noting that the SCWT scores were significantly associated with contralateral areas to the hand performing the task (i.e., L-PFC2 and L-PFC1 with Δ[HbR]-RG; R-PFC2 and R-PFC1 with Δ[HbR]-LG), while SCWE to the ipsilateral hemisphere (i.e., R-VIS1, R-PFC2 and R-PFC1 with Δ[HbR]-RG).

### 2.4. fNIRS Results’ Interpretation

In this study, we used fNIRS for an ecological assessment of cortical activation in PD patients at different stages. Patients performed the same motor grasping task, unveiling different activation patterns over both motor and non-motor networks. Even if their illness condition spanned from early to moderate, we found a significant difference between the ePD group and the mPD group and an association between the level of cortical activation with the clinical variables. The overall results suggest that PD motor impairment affects the activation level of motor areas, depending on the stage of the pathology, while the recruitment of non-motor areas follows different trends of activation.

Specifically, we first verified that (despite there being no significant differences in cognitive reserve and executive functions, as measured by the SCW test) the mPD group is associated with higher levels of motor impairment and disease progression, compared with the ePD group (Table 1). Then, considering the comparison of the ePD and mPD group-level activation maps, we found that the ePD group had greater levels of activation and inhibition across task conditions and ROIs. Specifically, the ePD group presented greater activation levels in the SMN and VIS areas, while the mPD group required increased activation of the PFC areas (Figure 3 and Table 3).

This result is also supported by the last ROI-CA analysis performed for all patients, which reported significant correlations of fNIRS data with the CRIQ, disease duration, the UPDRS, and the SCW performances in the PFC and VIS areas (Table 4). Indeed, these results indicate that a worsening of clinical conditions is associated with a lower activation and inhibition of these areas (i.e., positive correlations are mostly found for Δ[HbR], while negative correlations are mostly found for $\Delta \left[{\mathrm{HbO}}_{2}\right]$). This result is corroborated by partial correlations, separating the effect of age as the covariate, which showed almost no change in their level of statistical significance (Table 5), supporting a specific effect of disease severity.

## 3. Discussion

The ePD group presented greater activation patterns in SMN areas than the mPD group, according to both LG and RG tasks. Considering that both groups performed the motor grasping task correctly, the lower levels of activation in the mPD group may provide an overall index of loss of function. Indeed, it is known that cerebral perfusion and cerebrovascular reactivity of motor regions in PD are significantly correlated with disease severity [40]. As a consequence, the motor region in PD patients tends to hypoactivation, according to disease progression and the stage of PD progression over time. Interestingly, we noticed a unilateral hemispheric difference in L-SMN due to RG (i.e., significant differences were found only in the left hemisphere), while LG presented significant differences on bilateral SMN (Figure 3 and Table 3). Nevertheless, significant differences due to RG involved additional channels in L-SMN, compared with LG. This effect could possibly be due to the right-handedness of almost all patients (Table 1), causing a greater activation of L-SMN because of the dominant hand. In addition, no significant correlations were found for the LG condition for $\Delta \left[{\mathrm{HbO}}_{2}\right]$ across all clinical variables (Table 4 and Table 5).

Conversely, a higher PFC activation in the mPD group, compared with the ePD group, is in line with current fNIRS studies in PD, which have investigated PFC activation during walk conditions. The shared hypothesis is that PFC compensates for motor impairment and acts as an overall index of cognitive load. Among these works, Maidan et al. [16] indicated that PFC activation levels can distinguish between different typologies of FoG, as one of the most common disturbances in PD. The role of PFC as a motor-cognitive compensation mechanism was also confirmed in a successive work considering PD without FoG [17]. In addition, a recent study concluded that PD patients with FoG present a greater PFC activation, compared with healthy controls (HC) and PD without FoG, during a lower-limb motor task, and a positive correlation with the severity of FoG [15]. Usual and dual-task (DT) walking in patients with and without FoG were also assessed by Dagan et al. [18] and Orcioli-Silva et al. [19], respectively. Patients were evaluated before and after anti-Parkinsonian medication and the results provided additional insights into the interplay between PFC activation and gait parameters. Other fNIRS studies found an increase of PFC activation during walking, DT walking, and postural stability tasks with respect to HC [20,21,29]. Cornejo et al. [24] reported that walking at an externally paced rhythm can further promote PFC activity with respect to usual walking. Similarly, Maidan et al. [26] suggested that DT walking, in contrast with obstacle negotiation, causes different involvements of PFC in PD and HC. The same authors also performed a longitudinal randomized control trial that demonstrated that a combined virtual reality and a treadmill training program helped to reduce PFC activation during obstacle negotiation and DT walking [25]. Another study, by Hoang et al. [28], revealed reduced dorsolateral-PFC activity and better gait parameters after 5 weeks of an exercise-based training program.

Moreover, our results of increased PFC activation in the mPD group, compared with the ePD group, paired to a reduction in activation in the VIS and SMN areas, may be attributed to the loss of function of these areas associated with disease progression. Indeed, recent studies indicated that early PD patients, compared with HC, presented a reduction in functional connectivity and cerebral blood flow in the SMN, VIS1, and VIS2 areas. Therefore, it is proposed as prodromal biomarker of neurovascular coupling impairment and disease progression [41]. Moreover, other evidence from MRI biomarkers indicated that motor rehabilitation in PD promotes the restoration of movement automaticity and the involvement of a frontal-parietal compensatory mechanism [42].

Among the limitations of this study, we identified an unbalanced sample size in the ePD and MPD groups, which requires caution in the generalization of the results to a broader population. Moreover, the significant age difference between the groups is another potential issue, as it is known that normal aging affects the level of automaticity during the execution of sequential movements [43]. Nevertheless, in our ROI-CA, we noticed that a partial correlation between the cortical activation level of all patients with the clinical variables, using age primarily as a covariate, did not affect the significance level. Hence, in our study, we concluded that the disease stage represented the primary driving reason for cortical activation differences in the ePD and MPD groups.

## 4. Materials and Methods

### 4.1. Participants

This study considered the analysis of 39 PD patients (age 69.0 ± 7.64, 38 right-handed) who were enrolled in the baseline assessment of the SIDERA^B project [44]. The inclusion criteria were as follows:

- age between 18 and 85 years (adult and older adult);
- agreement to participate, with signature on the informed consent form;
- clinical diagnosis of PD, according to the Movement Disorder Society (MDS) criteria [45] and disease staging between 1.5 and 3 on the Hoehn and Yahr scale [46]

The exclusion criteria were as follows:

- presence of comorbidities that might determine clinical instability (i.e., severe orthopedic or severe cognitive deficits);
- overlapping between PD and other neurological pathologies or by PD with severe psychiatric complications, based on a pathological score in a screening test for cognitive impairment (Montreal Cognitive Assessment test—MoCA test < 17.54 [47])

Patients were subdivided into two different groups according to the different stage of the pathology, as assessed by the Hoehn and Yahr (HY) scale. Specifically, patients with HY scores of 1 or 1.5] were assigned to the ePD group, while patients were assigned to the mPD group if they had HY scores of 2, 2.5, or 3]. All patients provided their informed consent, and the study was conducted according to the guidelines of the Declaration of Helsinki and approved by the Ethics Committee of IRCCS Fondazione Don Carlo Gnocchi.

### 4.2. Clinical Charaterization and Neuropsychological Assesment

The overall patient demographics and clinical variables are reported in Table 6, while further details of the ePD group and. the mPD group are presented in the Results Section in Table 1. Among the clinical variables, we employed the MDS-UPDRS and disease duration to determine if the ePD and mPD groups significantly differed in terms of motor impairment, while the CRIQ [48] was used to verify whether the subgroups differed in terms of cognitive reserve and, hence, whether measured hemodynamic variations by fNIRS could be entirely attributed to PD pathology. Further variables, such as EQ5D5L [49] and education, were also employed for participants’ characterization.

Finally, a computerized version of the SCW test by Caffarra et al. [50] was administered as a neurophysichological test score to evaluate the activation of the subjects’ non-motor areas. This test was administered on the same day and in correspondence with the fNIRS acquisition (as described in Section 4.3). All patients were in the ON-state of the anti-Parkinsonian medications when the SCW test was performed.

This test was subdivided into three trials, indicated as color (C), word (W), and interference trials (CW). The former two trials required patients to read the name of color words (i.e, “red”, “blue”, and “green”) and to state the color of squared shapes. Conversely, in the CW trial, color words were dyed with a hue that did not represent the semantic meaning of the word, thus requiring the patients to state the hue instead of the color word (i.e., if the color word “red” was presented in blue ink, the participant had to respond “blue”). The computerized version required patients to respond to these trials by pressing three color-coded buttons. The resulting SCWE and SCWT scores were computed, respectively, as the difference between the number of errors or the time of the CW trial and the average of the C trial and the W trial.

All demographic data and clinical variables were tested for normality by the Shapiro–Wilk test for the whole group and for the ePD and mPD subdivisions in Jamovi 1.8.1.0 (the Jamovi project (2021), https://www.jamovi.org), allowing us to define appropriate statistical tests for the group’s characterization (Section 2.1) and to carry out ROI-based correlation analysis (Section 2.3 and Section 4.6). We also tested for significant differences in ePD and mPD group demographics and clinical variables, according to either an independent sample *t*-test or a Mann–Whitney U-test, depending on the data distribution (see Table 1 in the Results Section).

### 4.3. fNIRS Assessment

The experimental data were acquired from IRCCS Fondazione Don Carlo Gnocchi, Milan. We employed the NIRScoutX 32 × 32 (NIRx Medizintechnik, Berlin, Germany) to provide functional measurements at 760 and 850 nm. This continuous wave (CW) system used 32 LEDs sources and 32 avalanche photodiode detectors, which resulted in 102 measurement channels (i.e., source-detector combination pairs). The resulting sampling frequency was 1.9531 Hz. Sources and detectors were placed over the scalp positions of each subject, according to the International 10/5 system [51], thus identifying the channel positions as the midpoint between the source and detector positions (i.e., in the supplementary data at *optodeConfigurationROI* sheet, we state the correspondence between the source and detector number with International 10/5 locations).

In line with neuropsychological testing by SCW, all patients were in the ON state of the anti-Parkinsonian medications during the fNIRS acquisition. Because PD is a neurodegenerative disorder that is mainly characterized by motor deficits, a block-design motor-grasping task was employed to elicit the activation of the motor areas [52] and to observe ancillary activity in the non-motor areas. Participants were asked to sit still in a dimly lit room and to squeeze repeatedly two sponge balls that were placed within the palms of their left and right hands. A stimulus presentation PC running E-Prime 3.0 (Psychology Software Tools, Pittsburgh, PA, USA) was synchronized with the fNIRS system and placed in front of each participant. The resting condition was represented by a fixation cross placed in the center of the screen. The task condition involved the blinking at 0.8 Hz of the cross on either the left or right side of the screen, requiring participants to follow this blinking by pressing and releasing the ball with left or right hand. The stimuli presentation was repeated 10 times per side and randomized in left vs. right order. The task condition lasted 10 s and was followed by 20 s of resting.

Two experienced fNIRS users visually examined the performance of the participants to control the correct execution of the task, observing no major differences among them. No instrumental assessment of the motor performance was employed, as the task only required the participants to press and release a simple rubber ball.

### 4.4. Pre-Processing and Subject-Level Statistics of the fNIRS Data

**Figure 4:**
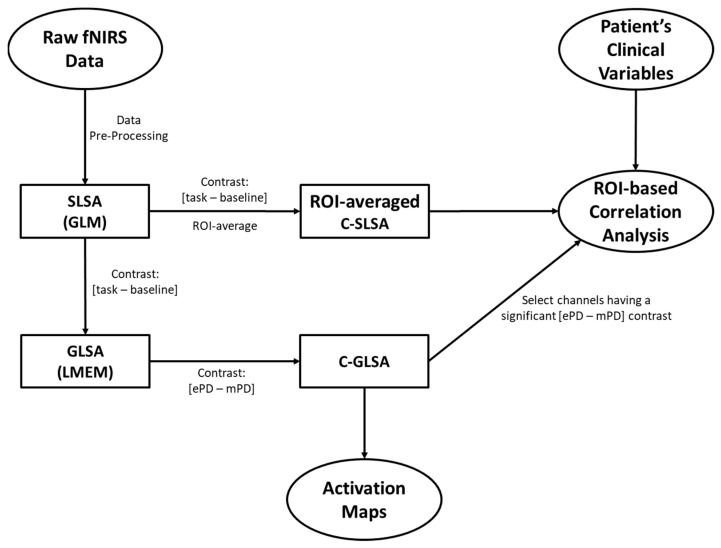
Conceptual framework of the main steps to obtain the ePD and mPD activation maps and perform ROI-based correlation analysis: subject-level statistical analysis (SLSA); group-level statistical analysis (GLSA); contrasted subject-level statistical analysis (C-SLSA); contrasted group-level statistical analysis (C-GLSA); general linear model (GLM); linear mixed effects model (LMEM).

A conceptual framework of the analyses performed in this work is provided in Figure 4. The pre-processing and statistical analysis of fNIRS data is still an open field in fNIRS literature and the procedure is non-standardized due to the heterogeneity of fNIRS applications, instrumentation, and experimental paradigms [53,54,55,56]. The estimation of fNIRS hemodynamic response assumes an even more important role when considering applications contextualized in clinical domains. In a previous work, we carefully considered these aspects by comparing the most-used artifact reduction algorithms in CW-fNIRS, without employing auxiliary sensing systems, as well as quantifying the reduction in signal energy at each step of a processing pipeline [57].

In this work, we translated our previous considerations over a clinical dataset. Data were analyzed in Matlab R2018b (The MathWorks, Inc., Natick, MA, USA) through user-defined scripts integrating Homer2 [58] and NIRS Brain AnalyzIR [59] toolboxes. Before data acquisition, two experienced fNIRS users verified that all channels presented a reasonable coupling with scalp positions. This information was provided as a quality measure of the signal-to-noise ratio by the acquisition software of the employed fNIRS system.

Then, raw data were converted into optical density variations, corrected by motion artifacts through a wavelet-based method [60], and high-pass filtered with a 0.01 Hz cut-off frequency. Then, we applied principal component analysis to remove up to 80% of data variance, thereby reducing the physiological interference derived from slow-varying oscillations and scalp-related superficial confounds, such as heart rate, Mayer waves, and respiratory oscillations [61,62].

We approached the subject-level statistical analysis (SLSA) of the fNIRS data according to the general linear model (GLM). The GLM parameter estimation required us to avoid low-pass smoothing filtering (added to the mentioned high-pass filtering) to prevent artifactual temporal correlations and to obtain white noise residuals [63,64]. We specifically employed the autoregressive iteratively reweighted least squares (AR-IRLS) by Barker et al. [65]. In brief, this algorithm adapts the traditional GLM analysis by iteratively estimating the coefficients of an AR(p) model (where p is the order of the model) to apply pre-whitening and reduce serially correlated errors, which are typical in fNIRS data. Additionally, the design matrix employed a constant regressor and the convolution between boxcar functions (the period is set as stimuli duration) and a canonical hemodynamic response function (cHRF) derived from fMRI literature [66] (with a maximum peak at 6 s). Convolution by the first derivative of cHRF was applied as a further regressor to account for the temporal dispersion of the real hemodynamic response, compared twitho cHRF.

Prior to group-level statistical analysis (GLSA), subject-specific GLM data were also channel-wise contrasted for task vs. resting conditions according to a two-tailed *t*-test. This approach allowed us to reduce the memory computation required by the group-level linear mixed-effects model (Section 2.4), while simultaneously controlling for the statistical significance of fNIRS data. Specifically, the 10 s LG and RG conditions were compared, respectively, to the following 20 s of resting periods. For the sake of simplicity, in the group-level analysis (Section 4.5), we indicated for LG and RG the contrast between task and resting conditions.

Resulting channel-wise positive $t_{\mathrm{value}}$ associated to p<0.05 indicated that activation occurred at that given channel, while negative $t_{\mathrm{value}}$ indicated inhibition. The false discovery rate by the Benjamini–Hochberg method was also used for multiple comparisons correction. 

### 4.5. Group-Level Statistics of fNIRS data

A linear mixed-effect model (LMEM) was computed for the GLSA of fNIRS data using NIRS Brain AnalyzIR toolbox. This model separately tested the effect of LG and RG conditions (i.e., brain activity) for each group, while controlling for subject as random effect. Accordingly, this model was expressed according to Wilkinson–Rogers notation as beta~−1+group:cond+(1|subject), where beta indicated the GLM regression coefficients deriving from SLSA, group referred to group membership (i.e., ePD vs. mPD), subject referred to the subject identifier, (1|subject) was a random intercept for each subject, and group:cond was the interaction between condition and group terms.

The results of the LMEM allowed us to compute three different typologies of group-level activation maps. First, we separately estimated the activation due to either LG or RG conditions in the ePD and mPD groups. Positive $t_{value}$ indicated activation, compared with the baseline condition (see the results in Figure 1 and Figure 2). Then, a two-tailed *t*-test contrast was applied to LG-ePD interaction vs. LG-mPD interaction, and similarly to RG-ePD interaction vs. RG-mPD interaction. Positive $t_{value}$ indicated that the mean activation level in the ePD group was higher than in the mPD group (see the results in Figure 3). In all cases, channel-wise activation was considered statistically significant if the resulting $t_{value}$ was associated with p<0.05. The false-discovery-rate method was still employed for multiple comparisons correction.

### 4.6. ROI-Based Correlation Analysis between fNIRS and Clinical Variables

Group-level activation maps allowed us to carry out numerical and visual evaluations of which channels were significantly activated or de-activated during LG or RG. A further refinement was provided by an ROI analysis addressing cortical areas, which required a correspondence between the whole-head channel configuration and functional-anatomical areas.

Specifically, the ROI analysis consisted of averaging the statistical results at SLSA and GLSA according to predefined lists of source-detector pairs placed in correspondence with functional-anatomical areas. We considered as functional cortical parcellations the bilateral Brodmann areas referred to the Colin27 atlas [67], due to its wide adoption in fNIRS applications [68] and software [9,58,59]. Specifically, we considered the composition of multiple bilateral Brodmann areas: BA1–2–3–4 as the primary sensorimotor network areas (left L-SMN and right R-SMN), BA17 and BA18–19 as the primary and secondary visual areas, respectively, in the occipital cortex (L-VIS1 and R-VIS1 vs. L-VIS2 and R-VIS2), and BA46–9 and BA45–47 as the high-level and low-level sites of executive functions in the dorsolateral- and ventrolateral-prefrontal cortex areas (L-PFC1 and R-PFC1 vs. L-PFC2 and R-PFC2).

We employed the Nirstorm package in Brainstorm [69] to virtually register the probe configuration in the Colin27 atlas. Then, we defined each ROI-specific list of source-detector pairs by computing the sensitivity profile of the channels to cortical anatomy via Monte Carlo simulations. Channels that presented a significant contribution to the mentioned cortical parcellations (i.e., channels that collected at least 20% of the ROI signal) were included in the respective ROI list of source-detector pairs. This analysis would possibly be applied to subject-specific anatomies to properly identify cortical parcellations referred to measurement channels. However, it is reported that an atlas-based approach still leads to reasonable results in image reconstruction when anatomical MRI is unavailable [70]. A summary table of functional vs. anatomical areas correspondence is provided in Table 7, while further information regarding source-detector correspondences to ROIs is provided in the supplementary materials (sheet *optodeConfigurationROI*). 

Finally, an ROI-based correlation analysis (ROI-CA) was conducted to identify whether there was a relationship between subject-specific cortical activations—as measured by GLM beta-coefficients from SLSA—and patient characteristics. More precisely, we were interested in assessing a relationship between the motor-grasping task with either motor regions (i.e., where we expected to find the most prominent activation) or non-motor regions (i.e., where we expected to find ancillary significant brain activity).

ROI-CA involved two successive steps. First, significant channels in the GLSA results, contrasted for differences in the ePD group and the mPD group, were noted for each selected ROI. Then, an ROI average was applied to the SLSA results, contrasted for LG and RG tasks and baseline by limiting the number of channels to the ones identified in the first step. This approach provided a data-driven method to restrict the correlation analysis of the fNIRS data for brain areas, primarily contributing to activation across all subjects and reducing the possibility of averaging non-significant activations at subject level.

We applied Spearman’s correlation in Jamovi 1.8.1.0, due to data distribution (see Table 1 in the Results Section), providing a $\rho_{S}$-correlation coefficient for each item of fNIRS data to the clinical variable relationship. Because we were interested solely in the association of patients’ characteristics with brain activity, we performed this ROI-CA for all subjects, including both the ePD group and the mPD group. Among the clinical variables listed in Table 6, we considered the correlation of fNIRS data with age, the CRIQ, disease duration, the UPDRS, the SCWE scores, and the SCWT scores. Indeed, the correlation with age, the CRIQ, disease duration, and the UPDRS allowed us to understand whether variations in the demographics and clinical outcomes were related to variations in the levels of brain activity. Conversely, the correlation of SCWE and SCWT scores provided more insights into significant ancillary activation of the non-motor areas. Finally, as age presented a significant correlation with both the motor areas and the non-motor areas, we also applied a partial Spearman’s correlation using age as a covariate, thereby noticing whether previous significant correlations were preserved.

## 5. Conclusions

This work may open new scenarios in the investigation of the progression of PD motor and non-motor impairment, using fNRIS as a non-invasive and ecological neurophysiological correlate of cortical activity. fNIRS data, if used in conjunction with current clinical outcomes, will provide viable support for the monitoring of disease progression and for the longitudinal assessment of therapeutic and rehabilitation procedures. In this study, we approached the aspect of PFC compensation for motor impairment from a different standpoint by using a grasping task instead of a walking task as in the current fNIRS literature. Data from our fNIRS signals show significant differences in the frontal-occipital regions and reveal that VIS areas are involved in this mechanism. Taken together, our results demonstrate that fNIRS could provide viable support for the longitudinal assessment of therapeutic and rehabilitation procedures and define new prodromal, low-cost, and ecological biomarkers of disease progression.

## Figures and Tables

**Table 1:** Patient’s characteristics and clinical variables with respect to the ePD group and the mPD group. Differences in participant characteristics were assessed either by (*) independent sample **t**-test or (**) the Mann–Whitney U-test, depending on data distribution. The clinical variables included the Cognitive Reserve Index questionnaire (CRIQ); the Hoehn and Yahr scale (HY); the Montreal Cognitive Assessment (MOCA); the Levodopa Equivalent Daily Dose (LEDD); the Unified Parkinson’s Disease Rating Scale (UPDRS); the Stroop Color and Word error score (SCWE); the Stroop Color and Word time score (SCWT); and the 5-level EQ5D5L scale (EQ5D5L). Bold highlights significant comparisons.

	ePD (N = 13) Mean (SE)	mPD (N = 26) Mean (SE)	ePD vs. mPD (dof, p)
**Age**	63.519 (1.6549)	71.676 (1.3652)	55.5 (37, <0.001) (**)
**Sex**	M/F 6/7 (46.15%)	M/F 14/12 (53.85%)	-
**Handedness**	R/L 12/1 (92.31%)	R/L 26/0 (100%)	-
**Education**	13.308 (1.0824)	11.423 (0.7425)	1.4508 (37, 0.155) (*)
**CRIQ**	131.385 (6.1899)	122.577 (3.8021)	1.272 (37, 0.211) (*)
**Disease duration**	2.903 (0.8005)	5.049 (0.4695)	**87** (37, 0.014) (**)
**HY**	1.423 (0.0521)	2.327 (0.0731)	-
**UPDRS**	16.077 (1.5626)	35.654 (2.3993)	**−5.4598** (37, <0.001) (*)
**LEDD**	405.385 (56.430)	538.961 (44.282)	1.797 (37, 0.08) (*)
**MOCA**	25.162 (0.798)	23.764 (0.536)	−1.479 (37, 0.148)
**SCWE**	9.083 (3.2543)	8.88 (1.6677)	132.5 (35, 0.58) (**)
**SCWT**	12.693 (2.4129)	18.716 (3.1002)	105 (34, 0.196) (**)
**EQ5D5L**	0.841 (0.0321)	0.754 (0.0248)	**96.5** (37, 0.031) **)

**Table 2:** Significant group-level results for the ePD and mPD groups across ROIs, task conditions, and chromophores. (*) significant results for $p_{FDR}<0.05$. Left hemisphere (L-); right hemisphere (R-); sensorimotor network (SMN); primary visual network (VIS1); secondary visual network (VIS2); dorsolateral prefrontal cortex (PFC1); ventrolateral prefrontal cortex (PFC2). Bold highlights significant comparisons.

ROI	Type	Contrast	Beta		T		*p*	
			ePD	mPD	ePD	mPD	ePD	mPD
**L-SMN**	$\Delta \left[{\mathrm{HbO}}_{2}\right]$	Left Grasp	0.046	−0.056	1.499	−2.748	0.136	0.007 (*)
	$\Delta \left[{\mathrm{HbO}}_{2}\right]$	Right Grasp	0.326	0.246	10.837	12.321	0.000 (*)	0.000 (*)
	Δ[HbR]	Right Grasp	−0.241	−0.203	−15.762	−22.111	0.000 (*)	0.000 (*)
**R-SMN**	$\Delta \left[{\mathrm{HbO}}_{2}\right]$	Left Grasp	0.283	0.229	8.972	13.543	0.000 (*)	0.000 (*)
	Δ[HbR]	Left Grasp	−0.203	−0.161	−13.815	−19.861	0.000 (*)	0.000 (*)
**R-VIS1**	Δ[HbR]	Left Grasp	0.007	−0.037	0.437	−3.348	0.663	0.001 (*)
	Δ[HbR]	Right Grasp	−0.033	−0.006	−2.046	−0.549	**0.043**	0.584
**L-VIS2**	Δ[HbR]	Left Grasp	0.009	−0.017	0.877	−2.518	0.382	0.013 (*)
	$\Delta \left[{\mathrm{HbO}}_{2}\right]$	Right Grasp	0.047	−0.037	2.112	−2.480	**0.036**	**0.014**
**R-VIS2**	Δ[HbR]	Left Grasp	0.013	−0.035	1.099	−4.558	0.273	0.000 (*)
**L-PFC2**	$\Delta \left[{\mathrm{HbO}}_{2}\right]$	Left Grasp	0.064	0.067	2.067	3.150	**0.040**	0.002 (*)
	Δ[HbR]	Left Grasp	−0.040	−0.017	−2.510	−1.647	**0.013**	0.102
	Δ[HbR]	Right Grasp	−0.049	−0.027	−3.106	−2.588	0.002 (*)	0.011 (*)
**R-PFC2**	$\Delta \left[{\mathrm{HbO}}_{2}\right]$	Left Grasp	0.014	0.037	0.464	2.024	0.643	**0.045**
	Δ[HbR]	Left Grasp	−0.062	−0.017	−4.678	−1.916	0.000 (*)	0.057
	$\Delta \left[{\mathrm{HbO}}_{2}\right]$	Right Grasp	0.080	0.033	2.653	1.823	0.009 (*)	0.070
	Δ[HbR]	Right Grasp	−0.067	−0.009	−5.069	−0.994	0.000 (*)	0.322
**L-PFC1**	$\Delta \left[{\mathrm{HbO}}_{2}\right]$	Left Grasp	0.042	0.030	2.107	2.245	**0.037**	**0.026**
	Δ[HbR]	Left Grasp	−0.029	−0.015	−3.008	−2.470	0.003 (*)	**0.015**
	Δ[HbR]	Right Grasp	−0.040	−0.029	−4.168	−4.732	0.000 (*)	0.000 (*)
**R-PFC1**	Δ[HbR]	Left Grasp	−0.047	−0.024	−5.297	−4.238	0.000 (*)	0.000 (*)
	$\Delta \left[{\mathrm{HbO}}_{2}\right]$	Right Grasp	0.045	0.018	2.253	1.402	**0.026**	0.163
	Δ[HbR]	Right Grasp	−0.034	−0.027	−3.905	−4.701	0.000 (*)	0.000 (*)

**Table 3:** Significant group-level contrasts between the ePD vs. mPD groups across ROIs, task conditions, and chromophores. Bold highlights significant comparisons.

ROI	Type	Contrast	Beta	SE	T	*p*	$p_{\mathrm{FDR}}$
**L-SMN**	$\Delta \left[{\mathrm{HbO}}_{2}\right]$	Left Grasp	0.102	0.037	2.768	**0.006**	**0.029**
	$\Delta \left[{\mathrm{HbO}}_{2}\right]$	Right Grasp	0.080	0.036	2.228	**0.027**	0.088
	Δ[HbR]	Right Grasp	−0.038	0.018	−2.120	**0.036**	0.103
**R-SMN**	Δ[HbR]	Left Grasp	−0.041	0.017	−2.452	**0.015**	0.054
**R-VIS1**	Δ[HbR]	Left Grasp	0.044	0.020	2.223	**0.028**	0.088
**L-VIS2**	Δ[HbR]	Left Grasp	0.026	0.012	2.112	**0.036**	0.103
	$\Delta \left[{\mathrm{HbO}}_{2}\right]$	Right Grasp	0.084	0.027	3.129	**0.002**	**0.012**
**R-VIS2**	Δ[HbR]	Left Grasp	0.048	0.014	3.377	**0.001**	0.006
**R-PFC2**	Δ[HbR]	Left Grasp	−0.046	0.016	−2.880	**0.005**	**0.022**
	Δ[HbR]	Right Grasp	−0.058	0.016	−3.706	**0.000**	**0.002**
**R-PFC1**	Δ[HbR]	Left Grasp	−0.022	0.011	−2.119	**0.036**	0.103

**Table 4:** Significant results of ROI-based correlation analysis between ROI averaged data and patient characteristics at subject level (*p* < 0.05). Results are provided by means of Spearman’s correlation coefficient $\rho_{S}$ ($p_{value}$). Bold highlights the significant comparisons.

Contrast	ROI	Age	CRIQ	Disease Duration	UPDRS	SCWE	SCWT
**Right Grasp** **Δ[HbO_2_]**	**R-VIS1**	0.077 (0.639)	−0.083 (0.617)	**−0.397 (0.012)**	−0.035 (0.832)	0.162 (0.338)	0.005 (0.976)
	**R-VIS2**	−0.156 (0.343)	−0.009 (0.956)	**−0.366 (0.022)**	**−0.388 (0.015)**	−0.063 (0.711)	−0.174 (0.311)
**Left Grasp** **Δ[HbO_2_]**	**-**	-	-	-	-	-	-
**Right Grasp** **Δ[HbR]**	**R-VIS1**	0.159 (0.334)	−0.159 (0.333)	−0.29 (0.073)	0.04 (0.81)	**0.371 (0.024)**	−0.047 (0.787)
	**L-SMN**	**0.441 (0.005)**	−0.059 (0.72)	0.241 (0.139)	0.22 (0.179)	−0.049 (0.773)	0.248 (0.145)
	**L-PFC2**	0.275 (0.091)	−0.027 (0.873)	0.05 (0.762)	0.142 (0.388)	0.245 (0.144)	**0.476 (0.003)**
	**R-PFC2**	**0.394 (0.013)**	−0.236 (0.149)	−0.056 (0.734)	0.192 (0.241)	**0.539 (<0.001)**	0.144 (0.403)
	**L-PFC1**	0.193 (0.239)	−0.1 (0.546)	0.006 (0.969)	0.208 (0.205)	0.057 (0.736)	**0.354 (0.034)**
	**R-PFC1**	**0.329 (0.041)**	−0.18 (0.274)	0.001 (0.998)	0.184 (0.263)	**0.464 (0.004)**	0.167 (0.33)
**Left Grasp** **Δ[HbR]**	**L-VIS2**	**−0.366 (0.022)**	0.259 (0.112)	−0.217 (0.185)	−0.19 (0.248)	0.13 (0.442)	0.138 (0.421)
	**L-SMN**	0.152 (0.356)	**−0.531 (<0.001)**	0.091 (0.583)	0.071 (0.666)	0.284 (0.089)	−0.057 (0.743)
	**R-SMN**	**0.345 (0.031)**	−0.137 (0.405)	0.242 (0.138)	0.185 (0.26)	0.132 (0.437)	0.184 (0.282)
	**L-PFC2**	**0.456 (0.004)**	−0.145 (0.377)	0.259 (0.111)	**0.335 (0.037)**	0.067 (0.693)	−0.016 (0.925)
	**R-PFC2**	0.115 (0.486)	0.01 (0.953)	0.138 (0.402)	0.208 (0.204)	0.252 (0.132)	**0.394 (0.017)**
	**R-PFC1**	0.047 (0.777)	−0.024 (0.886)	0.168 (0.307)	0.2 (0.222)	0.214 (0.203)	**0.398 (0.016)**

**Table 5:** Significant results of ROI-based partial correlation analysis between ROI averaged data and patient characteristics at subject level (p<0.05), using age as covariate. Results are provided by means of Spearman’s correlation coefficient $\rho_{S}\;\left(p_{value}\right)$. (*) Correlation values were statistically significant when not employing age as covariate. Bold highlights the significant comparisons.

Contrast	ROI	CRIQtotal	Disease Duration	UPDRS	SCWE	SCWT
**Right Grasp** **Δ[HbO_2_]**	**R-VIS1**	−0.064 (0.704)	**−0.41** **(0.011)**	−0.088 (0.6)	0.158 (0.359)	−0.011 (0.95)
	**R-VIS2**	−0.055 (0.742)	**−0.355** **(0.029)**	**−0.363** **(0.025)**	0.006 (0.971)	−0.147 (0.4)
**Left Grasp** **Δ[HbO_2_]**	**-**	-	-	-	-	-
**Right Grasp** **Δ[HbR]**	**R-VIS1**	−0.121 (0.468)	−0.314 (0.055)	−0.05 (0.765)	**0.347** **(0.038)**	−0.084 (0.63)
	**L-PFC2**	0.054 (0.748)	0.02 (0.903)	0 (0.999)	0.147 (0.391)	**0.448** **(0.007)**
	**R-PFC2**	−0.143 (0.392)	−0.11 (0.512)	−0.014 (0.932)	**0.457** **(0.005)**	0.073 (0.675)
	**L-PFC1**	−0.049 (0.771)	−0.016 (0.926)	0.128 (0.442)	−0.018 (0.919)	0.318 (0.063) (*)
	**R-PFC1**	−0.097 (0.561)	−0.039 (0.818)	0.017 (0.919)	**0.382** **(0.022)**	0.106 (0.543)
**Left Grasp** **Δ[HbR]**	**L-SMN**	**−0.515** **(<0.001)**	0.075 (0.654)	−0.008 (0.961)	0.259 (0.127)	−0.075 (0.67)
	**L-PFC2**	−0.022 (0.897)	0.236 (0.154)	0.131 (0.433) (*)	−0.105 (0.544)	−0.102 (0.559)
	**R-PFC2**	0.044 (0.795)	0.127 (0.447)	0.175 (0.294)	0.218 (0.202)	**0.381** **(0.024)**
	**R-PFC1**	−0.01 1 (0.947)	0.164 (0.326)	0.206 (0.215)	0.205 (0.231)	**0.399** **(0.018)**

**Table 6:** Patients’ characteristics and clinical variables.

	N	Mean (SE)	Skewness (SE)	Kurtosis (SE)	Shapiro–Wilk W (p)
**age**	39	68.957 (1.2226)	−0.538 (0.378)	−0.706 (0.741)	0.944 (0.051)
**sex**	M/F 20/19 (51.28%)	-	-	-	-
**handedness**	R/L 38/1 (97.44%)	-	-	-	-
**education**	39	12.051 (0.6212)	0.179 (0.378)	−0.389 (0.741)	0.94 (0.038)
**CRIQ**	39	125.513 (3.2905)	−0.439 (0.378)	−0.231 (0.741)	0.958 (0.154)
**disease** **duration**	39	4.333 (0.4371)	0.467 (0.378)	−0.223 (0.741)	0.964 (0.244)
**HY**	39	2.026 (0.0861)	0.162 (0.378)	−0.623 (0.741)	0.913 (0.005)
**UPDRS**	39	29.128 (2.2412)	0.574 (0.378)	−0.145 (0.741)	0.954 (0.114)
**MOCA**	39	24.23 (0.45)	0.088 (0.378)	−0.344 (0.741)	0.982 (0.768)
**LEDD**	39	494.436 (36.05)	0.839 (0.378)	1.565 (0.741)	0.947 (0.065)
**SCWE**	37	8.946 (1.5174)	0.922 (0.388)	−0.397 (0.759)	0.855 (<0.001)
**SCWT**	36	16.708 (2.2474)	1.995 (0.393)	6.544 (0.768)	0.844 (<0.001)
**EQ5D5L**	39	0.783 (0.0205)	−0.726 (0.378)	0.194 (0.741)	0.944 (0.051)

**Table 7:** Summary of employed left (L) vs. right (R) ROIs and their correspondence in functional and anatomical areas.

Functional Label	Functional Areas	Anatomical Areas
L-SMN/R-SMN	BA1–2–3–4	Sensorimotor Network
L-VIS1/R-VIS1	BA17	Visual Network 1 (Occipital Cortex)
L-VIS2/R-VIS2	BA18–19	Visual Network 2 (Occipital Cortex)
L-PFC1/R-PFC1	BA46–9	Prefronatal Cortex 1 (Dorsolateral)
L-PFC2/R-PFC2	BA45–47	Prefronatal Cortex 2 (Ventrolateral)

## Data Availability

The data are not publicly available due to privacy issues.

## Supplementary Materials

The following supporting information can be downloaded at: https://www.mdpi.com/article/10.3390/ijms232314897/s1.
